# Obituary

**Published:** 1871-01

**Authors:** 


### *Obituary—H. F. Chesbrough, M.D.*

Henry Freyer, second son of Ellis Sylvester and Elizabeth Ann Chesbrough, natives of Maryland, was born in Totness, Orangeburg District, S. C., Sept. 19, 1840. His father was at the time Engineer of the Louisville, Cincinnati and Charleston R. R., of which he remained in charge until its completion to Columbia, S. C. In 1842 the parents of Henry removed to the North, and in 1844 his father resumed the practice of his profession in Massachusetts and other New England States. The son received his primary school instruction in Stoughton and West Newton, Mass. He passed through one of the Grammar schools of Boston, and afterwards attended the Latin school of that city for three years, when in Jan., 1856, he went with his mother and brother to Chicago, his father having accepted professional employment there the year before. There Henry, (or Hal, as he was familiarly called by intimate friends,) passed through the High School, being the second to graduate in that institution. He then entered Yale in the class of '63, but owing to the asthma, with which he was quite early afflicted, the climate of New Haven could not be endured by him, and the faculty strongly urged him to enter Beloit College, Wis., which he did, and graduated in 1863. He then studied medicine, and received a diploma from Rush Medical College in 1865, and immediately entered the army as Ass't Surg. in the 153d Ill. Vols.

July 8, 1865, Dr. Chesbrough married Miss Mary H. Goodall of Beloit, Wis. In September his regiment was disbanded, and he went very soon after to New York, where he spent the season in attending medical lectures and visiting the principal hospitals of that city.

In June, 1866, he was appointed Surgeon of the Peninsular Division of the Northwestern R. R., and removed to Escanaba.

In Feb'y, 1868, he returned to Chicago, and commenced the practice of medicine in this city.

In May, 1858, the subject of this memoir became a member of the New England Congregational Church of Chicago, with which he remained connected until his death, which occurred Dec. 8, 1870. He leaves, besides his widow, one son, three years old.

Dr. Chesbrough has been known to the readers of this Journal by his well written clinical reports. His connection with the Dispensary department of Rush Medical College has been characterized by such assiduous attention, zeal and practical success, as alone to render his comparatively sudden removal by death, a loss deeply to be deplored by his coadjutors and the large number of patients to whom he had rendered himself both respected and beloved by his many noble qualities of head and heart. As a gentleman of refined manners, ripe education and culture, with a capacity and inclination for minute and careful study, and at the same time for grasping the broadest and most comprehensive ideas of Medicine as a science, we have rarely met his equal at his age. Alway urbane and courteous in his deportment, his bearing was but the exponent of his native kindliness and amiability of disposition.

Few men of his years had so truly achieved real success—not perhaps in pecuniary accumulation, extensive practice or general repute—but in laying broad and deep the sure foundation of future eminence.

His last illness which from the very commencement was attended with extreme suffering except while he was under the influence of narcotics, was borne with the most uncomplaining patience, and he met its finale with the firmness and resolution of a man and a Christian.

As a friend and professional associate we shall ever cherish his memory, whilst we extend to the immediate circle of afflicted relatives our most earnest sympathy.

[Through the kindness of Chas. T. Parkes, M.D., we are enabled to give the subjoined notes.—Ed.]

*Post mortem* examination of the body of Dr. H. F. Chesbrough, twelve hours after death. Made section of the abdomen only. Found the abdomen moderately distended; the peritoneal cavity containing a quantity of shreddy lymph; the usual evidences of general peritonitis were well marked. Found considerable congestion of the omental and intestinal vessels; the surface of the omentum, mesentery, and intestines were covered with quite a number of large patches of lymph; the under surface of the diaphragm was covered with a continuous layer of thick lymph; the intestinal walls were greatly discolored, and in places gangrenous disorganization had taken place; an attempt to lift up a fold of them would lead to a tear through the entire walls. No obstruction, perforation or intussusception was found. The other contents of the abdomen were apparently perfectly healthy.

---

The officers and attendants of the North Chicago Charity Dispensary met in the Library Rooms of the Rush Medical College, Dec. 10, 1870, and adopted the following resolutions:

*Whereas*, We have been called to mourn the death of Dr. Henry F. Chesbrough, Secretary of the North Chicago Charity Dispensary—

*Resolved*, That we have lost a faithful friend and helper.

*Resolved*, That the Dispensary has sustained the loss of a devoted and efficient officer.

*Resolved*, That we tender our heartfelt sympathy to the bereaved family.

*Resolved*, That these resolutions be sent to the Chicago Medical Journal and the city papers for publication.

Dec. 20, 1870. Curtis T. Fenn, *Sec. pro tem.*

